# Emerging biological insights enabled by high-resolution 3D motion data: promises, perspectives and pitfalls

**DOI:** 10.1242/jeb.245138

**Published:** 2023-02-08

**Authors:** Pauline Provini, Ariel L. Camp, Kristen E. Crandell

**Affiliations:** ^1^ Université Paris Cité, Inserm, System Engineering and Evolution Dynamics, F-75004 Paris, France; ^2^ Learning Planet Institute, F-75004 Paris, France; ^3^ Département Adaptations du Vivant, UMR 7179 CNRS/Muséum National d'Histoire Naturelle, F-75005 Paris, France; ^4^ Department of Musculoskeletal and Ageing Science, Institute of Life Course and Medical Sciences, University of Liverpool, Liverpool L78TX, UK; ^5^ School of Natural Sciences, Bangor University, Gwynedd LL57 2UW, UK

**Keywords:** History, Movement, Three-dimension, Motion capture, Kinematics

## Abstract

Deconstructing motion to better understand it is a key prerequisite in the field of comparative biomechanics. Since Marey and Muybridge's work, technical constraints have been the largest limitation to motion capture and analysis, which, in turn, limited what kinds of questions biologists could ask or answer. Throughout the history of our field, conceptual leaps and significant technical advances have generally worked hand in hand. Recently, high-resolution, three-dimensional (3D) motion data have become easier to acquire, providing new opportunities for comparative biomechanics. We describe how adding a third dimension of information has fuelled major paradigm shifts, not only leading to a reinterpretation of long-standing scientific questions but also allowing new questions to be asked. In this paper, we highlight recent work published in *Journal of Experimental Biology* and influenced by these studies, demonstrating the biological breakthroughs made with 3D data. Although amazing opportunities emerge from these technical and conceptual advances, high-resolution data often come with a price. Here, we discuss challenges of 3D data, including low-throughput methodology, costly equipment, low sample sizes, and complex analyses and presentation. Therefore, we propose guidelines for how and when to pursue 3D high-resolution data. We also suggest research areas that are poised for major new biological advances through emerging 3D data collection.

## Teaser

**Summary:** The development of high-resolution, three-dimensional (3D) motion data has sparked significant paradigm shifts in comparative biomechanics, enabling the emergence of new questions as well as the reinterpretation of long-standing scientific concerns.

## Introduction

Our field of comparative biomechanics has grown hand-in-hand with technological advances, allowing for new insights into existing structures, materials and motions. Central to the study of motion is the need to quantify it, facilitated by modern technical developments across platforms. Arguably one of the most influential advances in modern biomechanics has been the advent and growth of the ability to study organismal kinematics in three dimensions, facilitated by imaging. In this paper, we focus on ‘high-resolution’ three-dimensional (3D) data, defined here as (1) technical digital high-resolution, based on images with small (relative to the size of the organism) pixels, (2) image quality allowing for sufficiently high precision, accuracy and signal-to-noise ratio to fully capture the motion in question, and (3) based on sufficiently dense motion tracking to reconstruct the entire 3D structure, organism or group as it moves in all dimensions. We are not aiming to provide an exhaustive list of 3D kinematic analyses, but to propose examples that illustrate the different points of our reflection about what 3D kinematic data can bring to the field of comparative biomechanics.

### Motion in two dimensions

Étienne-Jules Marey and Eadweard Muybridge arguably first championed the study of motion with photography (Marey, 1874; Muybridge, 1887; for a review, see McHenry and Hedrick, 2023). Both developed imaging techniques that allowed sequential images in rapid succession, acting as the first definable studies of animal gait. Since that time, studies of locomotion with two-dimensional imaging have flourished. The relatively simple – compared with modern-day equipment – recording setup produced a profusion of data, from the field and the lab, on a variety of animal sizes. Collecting speed data, together with stride and step parameters (e.g. frequency, length, duty factor) clarified the effect of scaling between species (e.g. Abourachid, 2001; Biewener, 1982, 1983, 1990; Blob and Biewener, 2001; Gatesy and Biewener, 1991; McGowan et al., 2008) and within species (e.g. Main and Biewener, 2007). The observation and quantification of shifts in gait during avian flight (Spedding, 1986; Tobalske and Dial, 1996) or terrestrial locomotion (e.g. Druelle et al., 2021; Hoyt and Taylor, 1981; Maes and Abourachid, 2013; Nauwelaerts et al., 2013; Nyakatura et al., 2008; Schoonaert et al., 2016) provided important insights into the evolution of locomotion (e.g. Abourachid et al., 2019; Dial et al., 2015; Hildebrand, 1977). Measuring cranial kinematics of feeding in fishes led to a deeper understanding of the functional morphology (e.g. Alexander, 1967; Anker, 1977; Liem, 1967) and hydrodynamics of this complex system (Muller and Osse, 1978; Van Wassenbergh, 2015; Van Wassenbergh et al., 2006). It resulted in new theories about the evolution and modulation of specialist and generalist feeding behaviours (e.g. Liem, 1978, 1980).

Despite the unquestionable benefits arising from 2D kinematics analyses, applied to an impressive diversity of species and functions, some drawbacks exist. By definition, in the case of non-planar motion, direct quantification using pure 2D recordings becomes very difficult, often impossible. They are particularly frequent in complex, non-cyclical motions, such as grasping or prey capture. Specific set-up tricks can be used to address this problem. One of them could be to limit the motions the studied animal is able to perform to only allow for planar movements (e.g. by building a narrow walking track to only record straight gaits as in Verstappen et al., 2000). The diversity of behaviours that can be captured is therefore limited and their frequent occurrence in natural conditions can be questioned. In addition, many joint movements are 3D and cannot be accurately studied in 2D; therefore, 2D analyses tend to focus on whole-body motions, determining the motion of the centre of mass (e.g. Nauwelaerts et al., 2015; Nyakatura et al., 2012) or of a geometrical centre, derived from the collected images (e.g. Provini et al., 2012a, 2014), whereas relative or independent movements of a specific body part are more difficult to quantify.

### Motion in three dimensions

In the vast majority of cases, 3D information is measured by combining two or more 2D perspectives. Even if collecting images from more than one view facilitated the first 3D quantifications of motion, views were often taken asynchronously owing to technical limitations. For example, work exploring the function of the pectoral girdle in flight by Jenkins et al. (1988) combined a dorsal view and a latero-ventral view of a starling flying in a wind tunnel. Thanks to the cyclical nature of the avian wingbeat, the asynchronous data could be interpreted separately, and provided a detailed description of complex 3D motions, such as the furcula movements during flight. Similarly, the repetitive walking cycle of a quail or the cyclical paddling motions of a ringed teal allowed for the reconstruction of a frontal view, built from the temporal synchronisation of the lateral and dorsoventral views (e.g. Abourachid et al., 2011; Provini et al., 2012b). The stereotypic, cyclical and repetitive nature of locomotor movements perfectly fits these reconstruction methods. However, many natural motions are not predictable and repetitive cycles, for a myriad of reasons. These include moments of burst performance, isolated or brief behaviours, as well as variation in species age, abilities and health. To overcome this, what could be seen as a failure to record a clean movement sometimes happens to be useful. For example, when trying to quantify the oropharyngeal–esophageal cavity (OEC) volume in a white-throated sparrow, spontaneously singing in front of an X-ray camera (Riede and Suthers, 2009), the sudden and unexpected neck rotation, occurring during the production of a similar note, completed the information extracted from the pure lateral view and provided indispensable information to estimate the volume of the OEC.

To obtain synchronous views of the same movement, inclined mirrors were often used to split a single view into two. This technique was used with light-based video cameras in a complement of single-plane X-ray acquisitions, for example, to explore the respiration, eating and spitting motions of three-spined sticklebacks (*Gasterosteus aculeatus*) (Anker, 1977) or the locomotion of the lizard *Sceloporus clarkii* (Reilly and Delancey, 1997). Early stereophotography, combining two viewpoints, was used to quantify the wake of flying jackdaws (Spedding, 1986), and became a classical method to obtain 3D data (e.g. Ikeya et al., 2022). The idea of multiplying views to obtain several perspectives of the same object was pushed one step further with the design of advanced tracking devices (e.g. de Margerie, 2015, Décamps et al., 2017), adapted to motion capture in natural environments.

Extrapolating from two or more 2D viewpoints to reconstruct 3D data is notably different from the direct registration of 3D coordinates. Capturing multiple views synchronously has become easier over time, but combining those views into 3D information requires a significant effort. Dealing with calibration or distortion can be challenging, especially outside of laboratory conditions. Yet, these steps are indispensable to fully leverage the potential of 3D data, especially to reconstruct the 6 degrees of freedom of a structure of interest.

With technological advances, it is easier to collect and process high-resolution 3D data with small-pixel images (relative to the size of the organism), a high signal-to-noise ratio and enough markers to fully reconstruct the structure's shape and its motion in 3D. Many of these methods rely on tracking markers in each view to reconstruct the subject's 3D motion more rapidly. Depending on the imaging mode and equipment, these may include automatically tracking infrared-reflective (e.g. Pontzer et al., 2009; Warrick and Dial, 1998), radio-opaque (e.g. Brainerd et al., 2010) or active markers. Marker-tracking and 3D motion reconstruction is achieved through 3D motion capture (see Moeslund et al., 2006 for a summary of methods applied to human motion), or more generally using direct linear transformation (DLT) to track any kind of marker manually or automatically (Hedrick, 2008). Open-source versions of the DLT software (see Hedrick, 2008; Jackson et al., 2016; Theriault et al., 2014) have facilitated a burst of new 3D datasets, and additional techniques are now moving beyond markers to reconstruct 3D motion directly from silhouettes (e.g. Fontaine et al., 2009), and 3D temporal scanners that capture motion as a sequence of 3D meshes (Ruescas Nicolau et al., 2022).

*Journal of Experimental Biology* has been leading many of these breakthroughs in 3D kinematic analysis. In 2012, Theriault et al. (2014) reported that 70 papers, or 11% of *Journal of Experimental Biology*'s published content that year, relied on videos to measure kinematics. More recently, in 2021, that percentage has increased to 55 papers, or 14% of the publications in the *Journal of Experimental Biology*. Of those, 32 papers, or 8% of total papers, 58% of kinematics-specific papers reported three-dimensional kinematics (see McHenry and Hedrick, 2023, for more details). This paradigm shift in data collection has either allowed for new insights into old questions, which sometimes led us to update textbooks, or opened questions completely new to science. In the next section, we highlight three case studies, illustrating those scientific processes.

## Case studies

### New insight: ventilation and rib complex motions

**Fig. 1.**
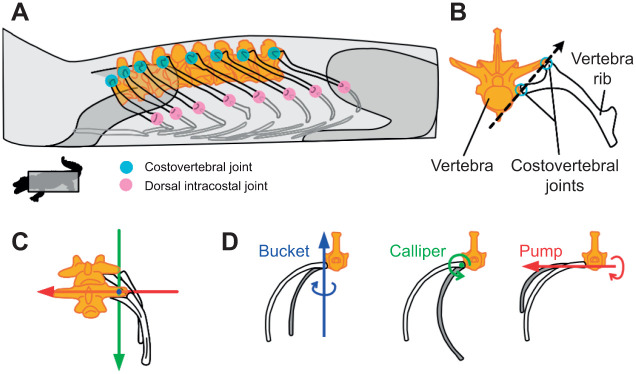
**New insights into rib ventilation in archosaurs.** (A) Anatomical diagram of the ribcage in an American alligator, including the vertebral column (orange), vertebral ribs (black outline), ventral and sternal ribs (grey outline), and costovertebral (blue circles) and dorsal intracostal (pink circles) joints (modified from Brocklehurst et al., 2017). (B) The bi-captiate morphology of the costovertebral joint was predicted to constrain this joint to hinge-like motion about a single, morphological axis (black dashed line). Figure redrawn from Hoffstetter and Gasc (1969). (C) Measurements of 3D costovertebral joint kinematics during breathing in live alligators using a joint coordinate system (JCS) (redrawn from Brocklehurst et al., 2017). (D) The JCS described the 3D, *in vivo* motion of the costovertebral joint along three axes of motion: ‘bucket-handle’ (rotation about the blue, dorsoventral axis), ‘caliper motion’ (rotation about the green, craniocaudal axis) and ‘pump-handle’ motion (rotation about the red, mediolateral axis). Redrawn from Capano et al. (2019). These measurements showed *in vivo* joint rotations deviated from the hinge-like rotation about the morphological axis.

The mechanics of breathing in crocodilians has been revisited in light of new observations coming from recent 3D motion visualisation techniques (Brocklehurst et al., 2017). Prior to X-ray reconstruction of moving morphology (XROMM) (Brainerd et al., 2010; Gatesy et al., 2010), 2D fluoroscopy was used to investigate ventilation in crocodilians (Claessens, 2004, 2009). Although able to quantify the relative contribution of the five mechanisms involved in crocodilian ventilation (e.g. pubic rotation, vertebral flexion, gastralial movement and, to a larger extent, costal aspiration and visceral translation) (Claessens, 2009), this method potentially missed fine movements of translations and rotations happening across joints during exhalation/inhalation. The expansion of the thorax, essential for costal aspiration, is associated with vertebral rib motions powered by intercostal muscles. The costovertebral joints – connecting the ribs to the vertebrae – were thought to behave like hinges (Claessens, 2009) (Fig. 1).

Surprisingly, a detailed 3D kinematics analysis of the costal aspiration of the American alligator (*Alligator mississippiensis*) (Brocklehurst et al., 2017) revealed a high degree of mobility of the intermediate ribs (Movie 1). The authors measured significant rotation about the dorsal intracostal joints with higher magnitude and complexity, especially in more caudal ribs, ruling out the ‘hinge model’ for crocodilians. The axis of rib rotation predicted by joint morphology, the ‘morphological axis’ (Fig. 1B) (Claessens, 2009), appeared substantially different from the *in vivo* joint rotations observed using high-resolution 3D techniques (Fig. 1C,D). Specifically, the morphological axis (Fig. 1B) underestimates bucket and overestimates pump motions (Fig. 1D). Considering the taxonomic position of crocodilians, generally used as an extant model for primitive archosaurs, this has consequences for the way we reconstruct the evolution of ventilation, one vital function in amniotes.

### Updating the textbooks: tongue motion

**Fig. 2.**
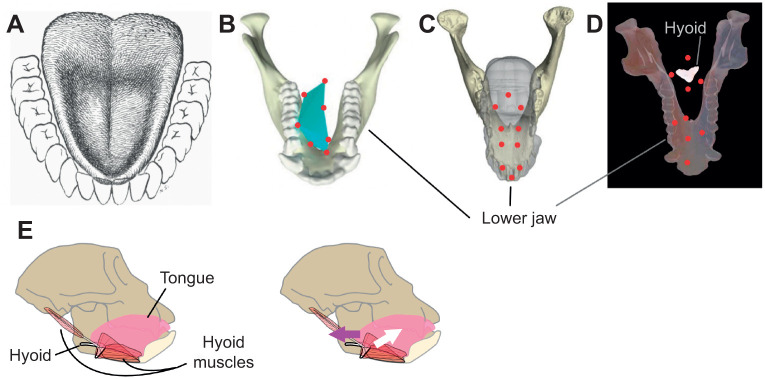
**Improved understanding of the mammalian tongue through 3D kinematic measurements.** (A) Early observations of tongue shape and deformation were limited to qualitative descriptions in subjects without teeth to obscure the view (image from Abd-el-Malek, 1955). (B–D) Biplanar X-ray video and implanted radio-opaque markers (red circles) allowed researchers to measure 3D, *in vivo* tongue deformation (turquoise shape shows reconstructed tongue surface) relative to the jaw (B,C) and hyoid (D) in macaques (*Macaca mulatta*) (B,D) and pigs (*Sus scrofa*) (C). (E) These 3D kinematic data have demonstrated the importance of the hyoid apparatus and the muscles acting on it (left figure). As the hyoid moves superiorly and anteriorly (white arrow), the base of the tongue moves posteriorly (magenta arrow) during swallowing in a macaque (right figure). Images modified from (B) Feilich et al. (2021) (https://creativecommons.org/licenses/by/4.0/), (C) Olson et al. (2021) and (D–E) Orsbon et al. (2020) (https://creativecommons.org/licenses/by/4.0/).

The mammalian tongue is a complex muscle and traditionally a textbook example of a muscular hydrostat, wherein the tongue is considered ‘incompressible’, such that shape change in one area causes compensatory shape changes elsewhere (Kier and Smith, 1985). Because the tongue is mostly located inside the buccal cavity during food processing, direct observations are difficult. Historically, the tongue's function during chewing in humans was investigated with subjects who lacked several teeth (Abd-el-Malek, 1955). However, considering the prominent role of the denture during mastication, this method came with limitations. With the increasing availability of fluoroscopy and the development of fluoromicrometry (Camp et al., 2016), radio-opaque markers helped to describe and quantify the complex motions (e.g. protraction and retraction) and complex deformation (e.g. changes in thickness) of the mammalian tongue (Fig. 2). Over time, the number of lingual markers increased (from 3 to more than 10), together with the frame rate and resolution of X-ray video recordings (Feilich et al., 2021; Olson et al., 2021; Orsbon et al., 2020). The high-resolution 3D data allowed for an accurate description and quantification of the tongue movements, as well as the relative sequence of motions of the jaws and hyoid (Hiiemae et al., 1995). Three-dimensional data have changed the way we see the system by adding a new actor – the hyoid – involved in tongue base retraction and the oral phase of swallowing (Orsbon et al., 2020) (Movie 2).

### Novel questions: coordinating flight manoeuvres

**Fig. 3.**
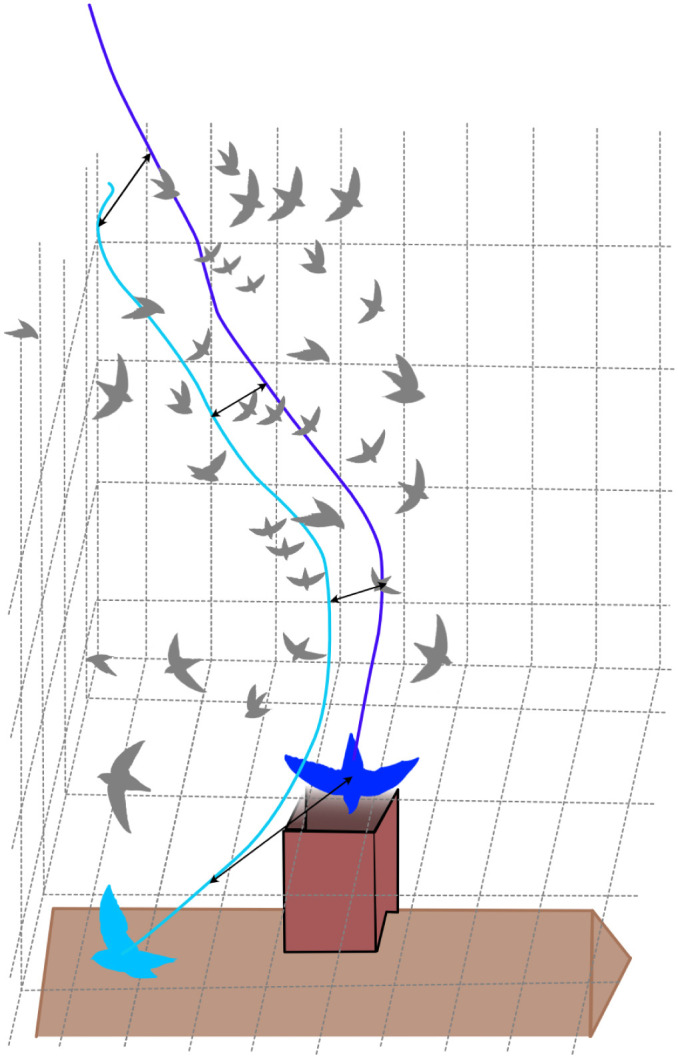
**Reconstructing 3D trajectories of chimney swifts to discover how they coordinate entry into a chimney roosting site.** Shown are the trajectories of two birds: one entering the chimney (dark blue line and bird silhouette) and one that did not enter (light blue line and bird silhouette), with the distances between these two birds shown with black arrows. Calculating these 3D trajectories allowed researchers to uncover how individuals interact within a flock. Figure modified from Parikh et al. (2019), bird silhouettes modified from an illustration by Gabriela Palomo-Munoz (https://creativecommons.org/licenses/by-nc/3.0/).

Motion within the 3D media of air and water has, by virtue of the complexities of the habitat, remained difficult to quantify – early explorations of animals moving in air and water were often limited to laboratory conditions, where motion patterns were kept relatively repeatable and orthogonal to the view. With the advent of accessible 3D tracking, both the media and the organism's motion within it can be quantified, and new work exploring motion within the natural environment has appeared. The resolution we are able to obtain has allowed us to address questions previously unattainable, including questions in the fields of animal behaviour and navigation. Recent examples explored whole-body trajectories during complex behaviours. Two such studies examined collision avoidance in roosting swifts (Parikh et al., 2019) and group behaviour during flocking (Evangelista et al., 2017) by collecting 3D kinematic data in a natural setting, using six synchronised high-speed light cameras and a calibration technique adapted to outdoor video recordings (Theriault et al., 2014). Chimney swifts roost communally, with hundreds of animals flying into a single roost site within a short timescale (Fig. 3). Parikh et al. (2019) discovered that during group landing events, animals coordinate landings by adopting slightly different approach angles and/or by following other animals closely. Work with this same species also established that birds relied on the physical distance to all neighbours during flocking flight (Evangelista et al., 2017) (Movie 3). Further work on flocking behaviour in jackdaws found that these same spacing rules change depending on circumstances – birds flocking in a straight line maintained physical distance between a set number of neighbours, regardless of distance from those neighbours. Birds flocking during a mobbing event maintained distance from all neighbours within a radius from themselves, which allows flocks to become more ordered as density increases (Ling et al., 2019). Without 3D tracking, the interactions between individual animals would be impossible to measure.

## Potential pitfalls of high-resolution 3D data

**Fig. 4.**
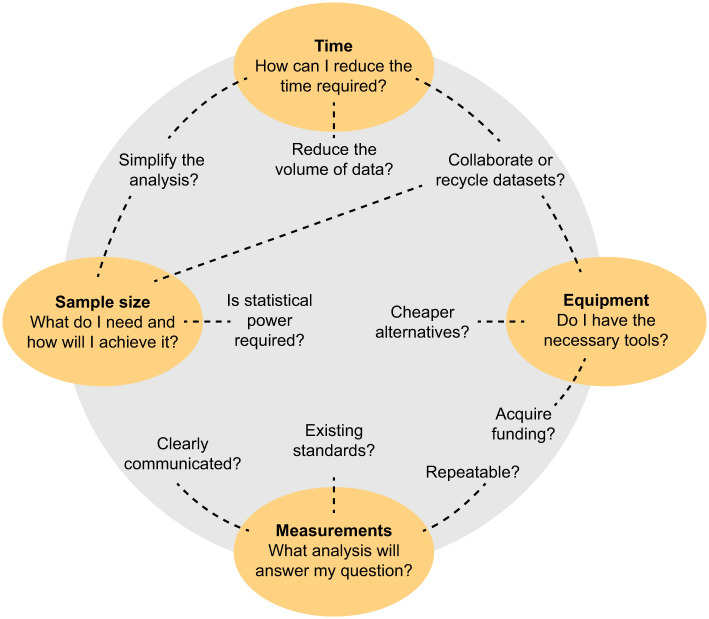
**Guiding questions to minimise pitfalls and maximise best practices methods of high-resolution 3D kinematics studies.** For each of the four pitfalls (highlighted in yellow), questions are suggested to guide researchers to identify the most appropriate solution for their study. We envision questions – to consider throughout the research process – to explore how to reduce the impact of these limitations on your study.

The examples above demonstrate the versatility and power of high-resolution 3D kinematics, but this method is not the answer to all questions about organismal motion. Below, we highlight four of the main limitations of high-resolution 3D techniques to provide a framework for deciding when these methods may – and may not – be useful. Although we suggest strategies and recent advances to minimise these limitations, there will always be trade-offs. Generally, the detailed depth of high-resolution 3D data comes at the cost of breadth of behaviours, replicates or species. Before embarking on a study, it is worth considering whether (1) a research question can only be answered with 3D kinematics and (2) how the limitations will be overcome (Fig. 4).

### Lengthy analysis

High-resolution 3D kinematics is rarely a high-throughput method. The rate-limiting step is usually the processing of images to extract 3D measurements, which can include calibration, marker tracking, marker identification and aligning morphological models to kinematic images. By comparison, modern recording equipment has made it dangerously easy to collect an enormous amount of data from more cameras, with high-resolution images, at high frame rates, over larger volumes, and with more markers. These large datasets demand substantial time to analyse, and excellent data management (Brainerd et al., 2017). Three-dimensional analysis methods have improved, e.g. computer-based calibration, tracking software, and automated or semi-automated tracking algorithms, but substantial time and expertise are still required especially for the highest precision and accuracy. This can make 3D kinematic studies expensive in terms of the time, computing power and staff required. We do expect analysis methods will continue to become faster and cheaper, with exciting developments including DeepLabCut (Mathis et al., 2018), Autoscoper (Miranda et al., 2011) and DANNCE (Dunn et al., 2021). Whether such analysis tools can enable most high-resolution 3D kinematics to be a high-throughput method remains to be seen.

### Expensive equipment

Collecting high-resolution 3D kinematic data often requires owning or accessing expensive equipment (tens to hundreds of thousands of pounds). Some of the most expensive examples are biplanar X-ray videography and video motion capture. These systems cost on the scale of hundreds of thousands of pounds to build and maintain, so accessing them as an external user may also be quite expensive. Such specialised equipment – like most 3D high-resolution kinematic data collection, regardless of cost – limits studies to behaviours recorded in unnatural or semi-natural lab environments (but see recent work in the field such as Clifton et al., 2015; Combes et al., 2012; Evangelista et al., 2017; Warrick et al., 2016). Looking forward, we expect the cost of 3D kinematic recording equipment to decrease and availability to increase. We are also encouraged to see cheaper methods (often <£1000) being developed to collect 3D kinematics. For example, PiROMM (https://doi.org/10.6084/m9.figshare.5155462.v2) and VROMM (Hoffmann et al., 2018; Jimenez et al., 2018) use the XROMM workflow but replace the two X-ray videos with two or more Raspberry Pi video cameras or standard light-video cameras, respectively. Similarly, smartphone-based 3D motion capture (e.g. Aoyagi et al., 2022; Reimer et al., 2022) avoids the need for costly motion-capture setups. Many of these take advantage of the increase in quality and decrease in costs of video cameras over the last 20–30 years. For example, a 0.065-megapixel high-speed camera in 1994 cost over 5 times that of a 12-megapixel camera in 2022 (E. Brainerd, personal communication). Often, a researcher's smartphone may have sufficient image quality, resolution and frame rate. However, there can be trade-offs in the ease of data collection and analysis. For example, less-expensive cameras may lack built-in software tools for essential steps such as multi-camera synchronisation, distortion correction or trigger controls to precisely start and stop recording. Therefore, it is worth considering whether the expertise to overcome these challenges is available, and whether the low cost is worth the increased time.

### Low sample size

Because of the time and cost to collect and analyse high-resolution 3D data, these studies are usually limited to low sample sizes. This can be a fatal obstacle for studies that require high statistical power to answer their research question, e.g. looking for relatively small (compared with the variation in the population) effects. It is also difficult to carry out comparative or evolutionary studies that would require high-resolution 3D kinematics from a relatively large (more than 5–10) number of species. Although as more studies are completed and made available, sample sizes can potentially be increased by ‘recycling’ data from previous studies. As the analysis time and equipment costs decrease, we expect it will be possible to increase sample sizes to some extent (see fig. 3 in McHenry and Hedrick, 2023). We do not believe current high-resolution, high-precision 3D kinematic approaches will become high-throughput methods for analysing hundreds of individuals, although we would be thrilled to be disproved.

### Complex analysis and communication

Once high-resolution 3D kinematics are recorded and analysed, reporting these measurements can be challenging. Compared with 2D motion analysis, 3D motion is more complex and difficult to visualise on 2D screens and pages. Although many fields have developed standard 2D methods and measurements, few exist for 3D kinematics outside of human biomechanics (Wu et al., 2002, 2005). As a result, it can be a struggle to make 3D kinematic results clear and reproducible. Three-dimensional kinematic data are often reported as translations and/or rotations about three orthogonal axes, relative to another structure. Because these axes can be defined in a multitude of different ways, the 3D kinematics of a limb or head can be measured in a nearly infinite number of ways. This makes it difficult to directly compare results from different studies. For example, multiple studies of the structurally and kinematically complex fish skull have measured the same motions about different axes and relative to different structures (Camp and Brainerd, 2014; Olsen et al., 2019; Whitlow et al., 2022). Very often, 3D kinematics data are recorded to ensure that the studied motion can be correctly projected and further analysed on classic 2D planes (e.g. lateral, frontal planes).

Although 3D motion will always be complex, we hope standardisation will make these datasets easier to understand and replicate. For example, Gatesy et al. (2022) proposed standard methods for measuring 3D posture and kinematics of the hindlimbs of archosaurs, and standards for measuring many body regions exist for 3D human kinematics (Wu et al., 2002, 2005). If research communities can create similar standards for other anatomical regions and taxa, this would greatly improve the reproducibility and clarity of 3D kinematic studies. However, there will always be exceptional structures or organisms that fall outside any standards – that is the delight of biological diversity.

## Future directions and promises of high-resolution data

### Three-dimensional motion of deformable structures

Adding a dimension has powerfully shifted our understanding of motion, informed old questions and opened new research areas as technology develops further. Soft, deformable tissues, such as muscle, tendon, skin and soft tissue, can now be visualised and quantified in 3D. Traditionally these have been difficult to study *in vivo*, owing to visualisation challenges and complex shape changes in 3D. Muscle fibres, for example, take on complex shapes so that shape, orientation and even function may change in different patterns at different locations within the same muscle. Simplified models using muscle length change as a proxy for force production are likely incorrect (Bishop et al., 2021). Now, we are starting to visualise the underlying muscle shape changes in living animals with X-ray video (Camp et al., 2016), magnetomicrometry (Taylor et al., 2021) or 3D ultrasound (e.g. Lopata et al., 2010; Genna et al., 2021). Skin, too, changes shape and material properties when in use – a dramatic example being the wing skin of bats, which can change tension during a single wingbeat to alter the aerodynamic properties of the airfoil (Cheney et al., 2022).

Three-dimensional datasets from a variety of sources are being used to study how biological structures deform. X-ray computed tomography provides detailed visualisation of structures and can even quantify changes in shape and function. The pitcher plant *Nepenthis gracilis* deformation was recently digitised using microtomography, illustrating how the lid deformation in 3D contributed to jerk forces necessary to capture prey (Lenz and Bauer, 2022). Three-dimensional models created using stereo imaging correlation (3D-DIC) tracked Venus flytrap opening motions, exploring how smooth bending, followed by a snap in some species, re-establishes the open trap (Durak et al., 2022).

### Recycling 3D data for new, comparative questions

The combination of detailed datasets with increased digital accessibility enables a single kinematic dataset to contribute to studies well beyond its initial research program. Reuse of kinematic datasets is a growing possibility, thanks to databases of both raw and processed data, increased adoption of open-access policies, and good data management (Brainerd et al., 2017). ‘Recycling’ existing datasets to examine new research questions facilitates new research areas while avoiding additional protocols, time and expenses. For example, Evangelista et al. (2017) and Parikh et al. (2019) use the same dataset to address different questions, while the dataset from Camp et al. (2015) was reused in two new analyses (Camp and Brainerd, 2015; Olsen et al., 2017). Although such recycling – even within research groups as in the examples above – requires meticulous metadata, well-documented data storage and methods, and open, well-organised repositories (e.g. XMAPortal), the potential payoff is high. We encourage future studies to incorporate plans to maximise the longevity, discoverability and accessibility of their dataset.

Existing studies can also contribute to wider-scale comparative work (Brainerd et al., 2017). As previously discussed, comparative work in biomechanics has traditionally been difficult. Owing to the time-demanding nature of obtaining the datasets, studies were limited to fewer than 3–5 species (such as Provini and Abourachid, 2018 and Crandell and Tobalske, 2015). However, as more studies are carried out, there is the exciting possibility of combining existing studies to create a comparative dataset with a larger sample of individuals and/or species. This requires good data management for storing datasets and making them discoverable and accessible to future collaborators. Recently, this has expanded owing to readily accessible past datasets and the rapidly advancing automation strategies to digitise kinematic data (such as Clifton et al., 2020; Young et al., 2022; Zhan et al., 2021). We expect increased automation and accessibility will continue to facilitate comparative work.

### Pairing 3D kinematics with other datasets

Unique kinematic datasets can now be complemented by different types of high-resolution data, including functional morphology databases, phylogenies, ecologies and genomic data. When combined, these can provide insightful answers to questions about ecology and evolution. Large-scale functional morphology datasets (e.g. Bardua et al., 2021; Brosse et al., 2021; Evans et al., 2021; Felice et al., 2019) are ripe for further kinematic exploration. Combining multiple types of data across disciplines is becoming easier owing to the digitisation of this data. For example, digital archives join morphological and ecological data across species – AVONET for birds (Tobias et al., 2022), FISHBASE for fish (Froese and Pauly, 2000) and even Sharkipedia for elasmobranchs (Mull et al., 2022). More specialised databases, such as XenoCanto for vocalisations in birds and Watkins Marine Mammal Sound Database, exist as well. A natural next step would be to identify groups of interest for future kinematic studies by cross-correlating different existing data types. When combined with detailed phylogenies facilitated by next-generation DNA sequencing (such as Prum et al., 2015), future studies are well positioned for detailed comparative work across species. As historical museum collections continue to evolve beyond specimen-based collections to incorporate digital next-generation sequencing and morphological data, they will facilitate rapid digital cross-correlation between data types (Muñoz and Price, 2019). Museums remain crucial to serve as a repository and hub for bringing together different research communities to create new datasets.

## Conclusions

We hope that the future of 3D kinematic studies continues to flourish with the advancement of technology, and would encourage future research to prioritise both realistic data collection practices as well as incorporate best practices to maximise data longevity, with a focus on repeatability, meticulous metadata and accessible archiving. With care in planning our data collection techniques, modern 3D data collection and analysis techniques will continue to illuminate the motions around us for years to come.

With the advancement of 3D analysis capabilities, new questions are now testable, allowing us to both update our knowledge and fill in the ‘blind spots’ in comparative biomechanics: from breathing in crocodilians to exploring mammalian tongue dynamics, quantifying aerial flight manoeuvres, and well beyond. As 3D motion better represents ‘real-world’ conditions, it will directly input toward building a better understanding of form–function relationships in the field of biomechanics. The reach of these datasets can go beyond our biomechanics niche – with applications to physical, digital and robotic models within health, industry and teaching.

## Supplementary Material

10.1242/jexbio.245138_sup1 Supplementary information
