# Supplementary material for: Emerging biological insights enabled by high-resolution 3D motion data: promises, perspectives and pitfalls
Source: J Exp Biol. 2023 Feb 8;226(Suppl 1):jeb245138. doi: 10.1242/jeb.245138 (PMC10038148; doi:10.1242/jeb.245138)
Supplement: Supplementary information [file jexbio-226-245138-s1.pdf]

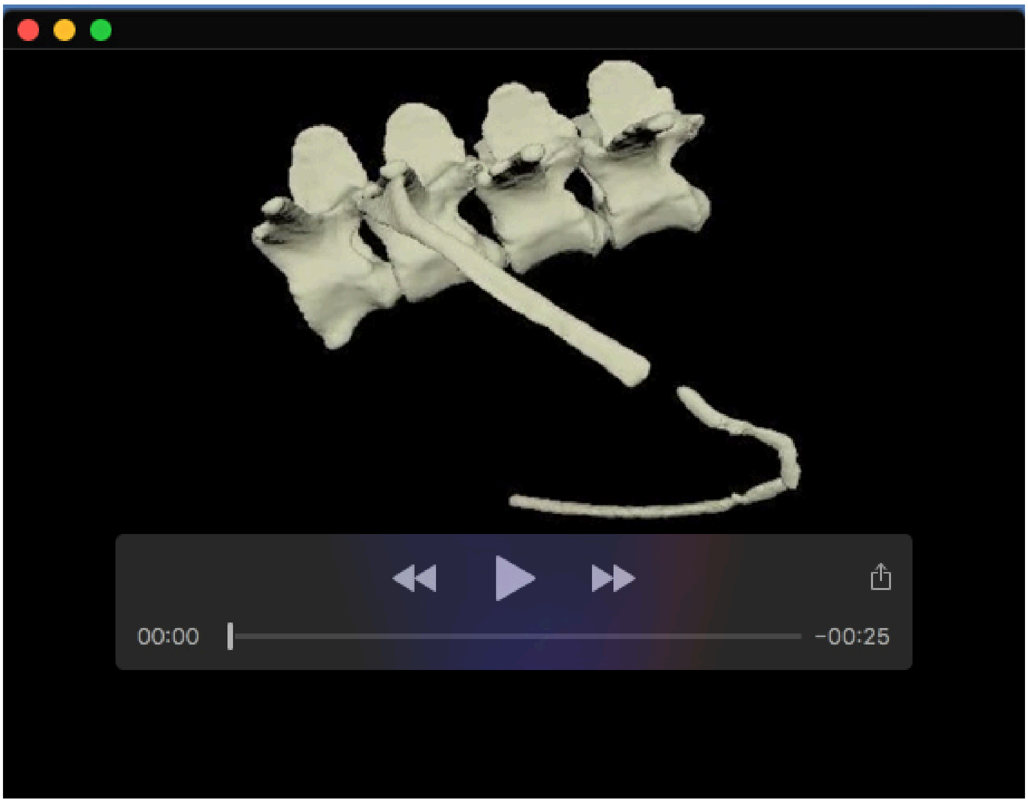

Movie 1. XROMM animation of ventilatory rib kinematics in American alligator (*Alligator mississippiensis*). The fourth rib (including the vertebral, ventral, and sternal ribs) and 3rd-6th thoracic vertebrae are shown in a lateral and then dorsal view (cranial is to the left). Video from (Brocklehurst et al., 2017) (http://creativecommons.org/licenses/by/3.0).

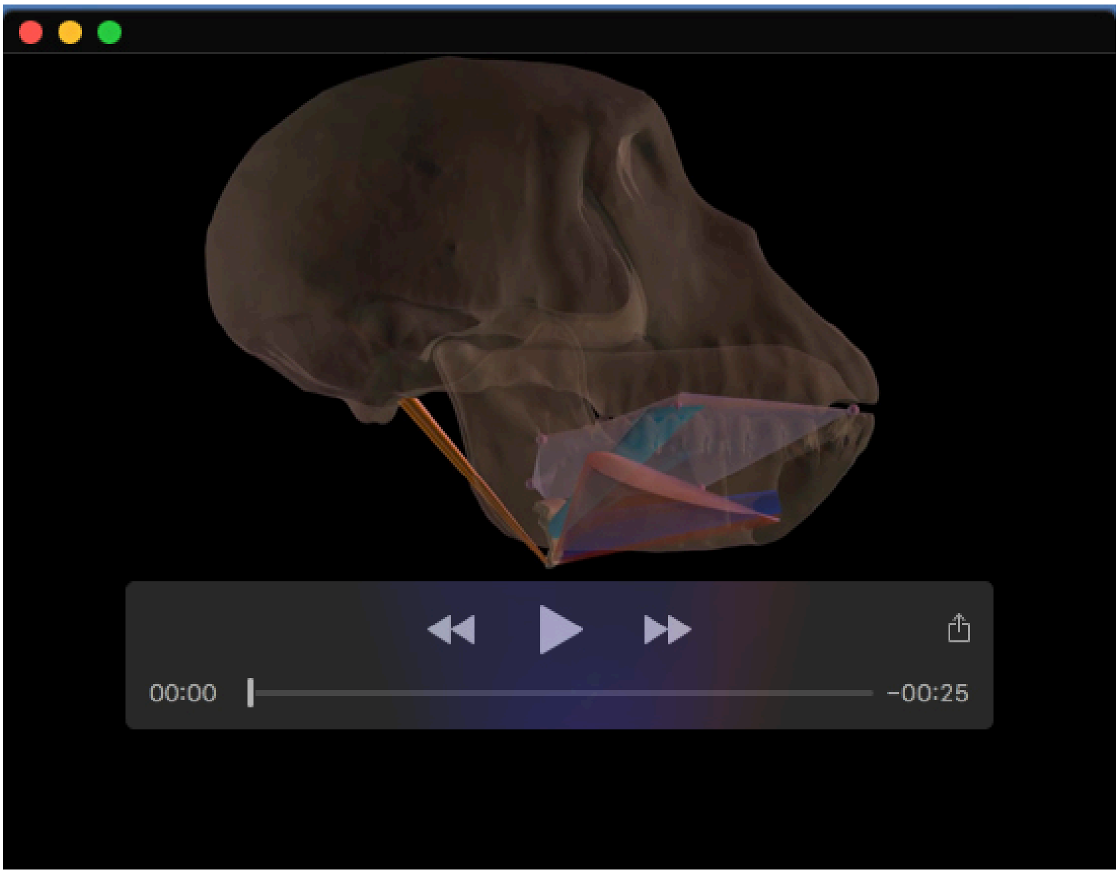

Movie 2. XROMM animation of the mandible, hyoid, and tongue, and reconstructed muscle length changes, relative to the cranium, during chewing in a macaque (*Macaca mulatta*). Right-lateral view of the head, including the bones (grey), tongue fluoromicrometry markers (pink spheres), reconstructed tongue volume (semi-transparent pink), posterior digastric muscle (orange), mylohyoid muscle (red), geniohyoid muscle (dark blue), and styloglossus muscle (cyan). Video from (Orsbon et al., 2020) http://creativecommons.org/licenses/by/4.0/

Journal of Experimental Biology • Supplementary information

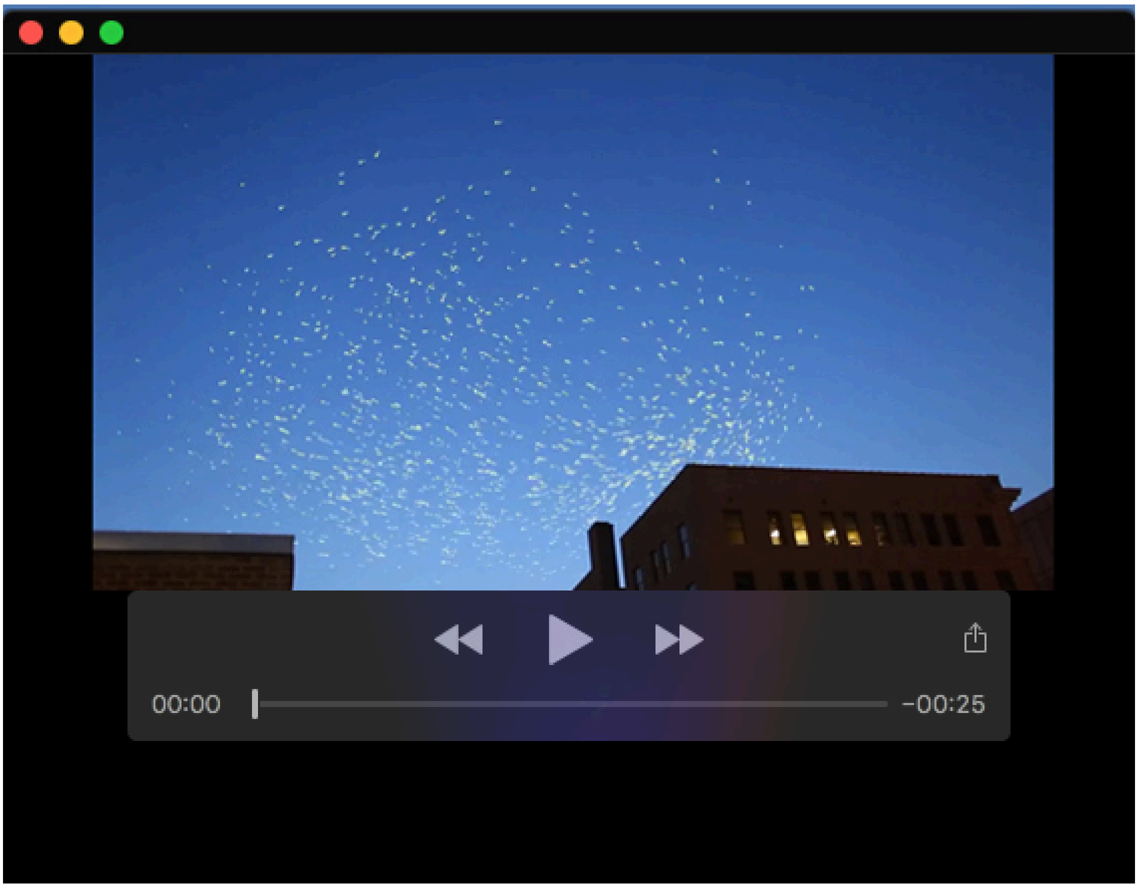

Movie 3. Roost entry of a chimney swift landing flock. Individual birds are highlighted in yellow, with larger profiles near the chimney roost (bottom right of the flock) as they slow down and extend the wings. Video from (Evangelista et al., 2017) (https://creativecommons.org/licenses/by/4.0/)
